# Supporting activity engagement by family carers at home: maintenance of agency and personhood in dementia

**DOI:** 10.1080/17482631.2016.1267316

**Published:** 2017-01-06

**Authors:** Pat Yin Fan Chung, Caroline Ellis-Hill, Peter Coleman

**Affiliations:** ^a^ Occuaptional Therapy Pathway, School of Allied Health Professions, Canterbury Christ Church University, Canterbury, UK; ^b^ Faculty of Health and Social Sciences, Bournemouth University, Canterbury, UK; ^c^ Faculty of Social & Human Sciences, University of Southampton, Southampton, UK

**Keywords:** Community-based practices, caregiving, dementia, person-centeredness, dementia-friendly communities

## Abstract

An explorative paper to describe how family carers, through the caregiving journey, reaffirm and promote the agency of people with dementia. Agency is an important concept in dementia care and is crucial to the promotion of wellbeing and the delivery of person-centred care. This article is based on one of the key findings of a study that explored family carers’ experiences of engaging their relatives in daily activities in domestic settings. Following research governance and ethical approval, 30 in-depth interviews (initial and follow-up) were carried out with 15 resident-carers of people with dementia who were recruited via local community mental health teams. Then five focus groups were conducted with 21 participants accessed through carers support groups. Interviews and focus groups were transcribed, coded and analysed using a grounded theory method. Findings showed the process in which family carers encouraged and sustained a sense of autonomy and control (agency) in their relative’s daily activities. Key strategies used by carers included: being non-judgemental; facilitating a sense of worth; taking calculated risks; maintaining the continuity of their relative’s identity; enhancing a sense of connection with their relative’s role and identity using enjoyable activities; preventing inactivity and attending to the bodily source of the agency. Lack of support for carers could ultimately pose a risk to the maintenance of the agency of people with dementia. This study provides a deeper insight into the process used by home carers to support the agency of people with dementia. This is essential if practitioners are to identify and develop more realistic intervention strategies and to work in effective partnership with family carers. The implications for the creation of dementia-friendly communities are discussed.

## Introduction

Dementia is a syndrome that can be caused by a variety of progressive illnesses that affect memory, thinking, behaviour and the ability to perform everyday activities (Alzheimer’s Disease International [ADI], 2012). Most subtypes of dementia are considered progressive in nature and cannot be cured by medication (Alzheimer’s Society [AS], 2007; Gubrium, 1987). Dementia is a major cause of dependency and disability (ADI, 2014). Increasingly, both national and global policies call for a shift in the way society supports people with dementia. This is to ensure, firstly, that they are enabled to continue to engage in everyday activities as long as they possibly can and, secondly, that they are prevented from going into institutional settings prematurely (UK Prime Minister’s Challenge on Dementia 2020; Department of Health [DH], 2012, 2015; World Health Organisation [WHO], 2012). Such a policy trend is in line with a person-centred approach, which is commonly considered by many countries as good practice (Behuniak, 2010; Edvardsson, Winblad, & Sandman, 2008; Robinson, Bamford, Briel, Spencer, & Whitty, 2010; WHO, 2012). Using this approach people are seen not only with a neurological focus, but also with recognition of their past experience, personality, current strengths, abilities, psychosocial needs, wishes and preferences. In the UK, government policies (e.g., National Dementia Strategies, the Prime Minister Challenge on Dementia 2020) reinforce the need to support individuals to be able to live well with dementia and experience wellbeing despite cognitive impairment (e.g., DH, 2009, 2015). In the long-term, the opportunity to participate in everyday activities is crucial in maintaining the personhood and a sense of agency (sense of control) of those with dementia. Family carers are key in supporting their relatives to engage in these activities. Family carers have been recognized as “partners” in care in government policies (DH, 2008), and it is important to understand their viewpoint. This article is based on the findings of a larger study that aimed to explore carers’ perceptions of how they have made decisions regarding their engagement with their relative in everyday activities at home.

Kitwood (1993) considered a person with dementia as an agent, like all of us, and as “a sentient, relational and historical being” (p. 583), capable of taking control of his or her personal life in a meaningful way (Kitwood & Bredin, 1992). Such a person can make things happen in their personal environment. The maintenance of agency is closely related to the wellbeing and personhood of those with dementia (Kitwood, 1993). Kitwood attributed personhood to individuals with dementia and defined the term personhood as “a standing or status that is bestowed upon one human being, by others, in the context of relationship and social being. In his view, personhood implies ‘recognition, respect and trust’” (Kitwood, 1997a, p. 8). Central to personhood is the recognition that the person with dementia has a self with a range of psychosocial needs including occupation, attachment, comfort, identity and inclusion (Kitwood, 1990). Kitwood (1997a) argued that the ability of the person to maintain the self, personhood and his/her psychological needs is not just reliant on his or her cognition. Those around this individual have a moral duty to support his or her personhood and the self. Similarly, Sabat (2001), exploring the selfhood of people with Alzheimer’s disease, concluded that certain aspects of the self persist. Such aspects include “personal identity” (p. 276), “mental and physical attributes” (p. 290) in the past and present and “socially presented selves/social identity” (p. 294). Nevertheless, he pointed out that the social self is particularly vulnerable because it is often dependent on the support of others. Hence, interdependent relationships between the person and others can both positively and negatively impact his/her personhood and sense of confidence. Despite memory difficulties, people continue to have the desire to communicate and connect with others. Jaworska (1999) and Jennings (2009) contended that those with dementia continue to participate in “meaning-sending and meaning-receiving relationships” (p. 430) with those around them through verbal and non-verbal means, despite severe decline in cognitive functioning (semantic agency). Hence, both agency and personhood can be nurtured with a supportive person-centred caregiving environment and moral relationships in daily life.

As discussed earlier, occupation is considered to be an important need for all people, including people with dementia, and is crucial to personhood because it provides an opportunity for positive interaction, attachment and inclusion (Kitwood, 1997a). Wilcock (2001) claims that humans have an “innate need to engage in occupation” (p. 5) leading to the maintenance of health and wellbeing within one’s sociocultural context (Wilcock, 1995, 2001). Through engaging in meaningful activities, individuals can gain a sense of choice, commitment, positive meaning and interaction (European Network of Occupational Therapy in Higher Education, 2010). Csikszentmihalyi (1988, 1993) argues that a meaningful life depends on an individual’s ability to find occupations that are sufficiently challenging within the scope of one’s capabilities. As dementia progresses, individuals often experience difficulty in coping with daily activities (Andersen, Wittrup-Jensen, Lolk, Andersen, & Kragh-Sørensen, 2004; Nygard, 2008; Phinney, 2008). Without appropriate support to address their activity needs, people may resort to communicating their unmet needs through the manifestation of psychological and behavioural symptoms, including restlessness, wandering, depression, mood change and agitation.

In England, the need to promote the use of activity-based interventions as a non-pharmacological approach has been considered as the first line of intervention strategy for managing cognitive, behavioural and psychological dementia symptoms (ADI, 2012; National Institute for Health and Clinical Excellence, 2006/2016). This results from an increasing acknowledgment of the side-effects of psychotropic medications on people with dementia (Livingston, Johnston, Katona, Paton, & Lyketsos, 2005). Most people with dementia are cared for by their family, who play an important role in supporting them in the management of everyday activities, despite the disabling effects of dementia (ADI, 2012; Perneczky et al., 2006). Moreover, as dementia progresses, the need to engage people with dementia in everyday activities at home often presents family carers with a challenge. Carers often report an ongoing sense of inadequacy and inability to manage their relative’s everyday activity (e.g., Keller, Edward, & Cook, 2007), affecting the carers own health and wellbeing (Smith, Williamson, Miller, & Schulz, 2011). These are the key factors affecting their decision to admit their relative to a care home (Gaugler, Duval, Anderson, & Kane Robert, 2007). If the implementation of government community-based policies is to be successful, it is necessary to understand how carers can be supported.

Past researchers into family caregiving activities have focused on general aspects of caring rather than on the need to engage their relative with dementia in activity. General aspects of caring include aspects such as respite (Arksey et al., 2004; Mason et al., 2007; Stirling et al., 2012), information (Thompson et al., 2007; Wald, Fahy, Walker, & Livingston, 2003) and psychosocial support/interventions (Brodaty, Thomson, Thompson, & Fine, 2005; Moniz-Cook et al., 2008; Pinquart & Sörensen, 2006; Selwood, Johnston, Katona, Lyketsos, & Livingston, 2007; Smits et al., 2007). Little is understood about how family carers support the wellbeing of their relative though engaging them in everyday activities. The aim of this article is to discuss one of the key themes emerged from the main study: to show how family carers recognized the continuing needs of their relatives and so enhanced their relative’s agency and autonomy, despite the progressive decline in cognitive function.

***Methods***

Given that little is known about issues concerning the topic under study—that is, how family carers perceive the use of everyday activities to maintain the wellbeing of people with dementia and themselves—a grounded theory method was adopted to facilitate the development of an explanatory theory of the basic social process of carers’ perceptions of the activity needs of people with dementia at home (as opposed to institutional settings). Grounded theory was initially developed by Glaser and Strauss in the 1960s. In the next few decades, they both acknowledged some differences between their versions of grounded theory (Glaser, 1978, 1992; Strauss & Corbin, 1998). Strauss and Corbin’s (1998) version of grounded theory method was particularly relevant to conduct this study, for it has its theoretical roots in pragmatism (Strauss & Corbin, 1998), which assumes that “meanings emerge through practical actions to solve problems, and through actions people come to know the world” (Charmaz, 2006, p. 188). They stressed the importance of studying process, action and meaning (Charmaz, 2006; Strauss & Corbin, 1998). This study addressed these three aspects as the researchers explored the processes by which carers of people with dementia engaged their cared-for relative in everyday routine activities over time. This involved listening to carers’ narratives of their actions and the meanings they attributed to their actions in relation to supporting their relatives in everyday activities.

### Participants

In the larger study, participants were co-resident carers who had specific experience of caregiving and responsibilities for their relative. An open sampling method (Hallberg, 2006, p. 143) was used for the two-phase study, seeking to maximize variations in experiences and descriptions by using participants from contrasting milieus and backgrounds. Phase 1 included carers who were living and looking after their relative with a diagnosis of dementia for at least 2 years; whereas Phase 2 included both carers who were still living and caring for their relatives and those who had become ex-carers. In order to ensure that these participants had experience of caring for a relative with a confirmed diagnosis, invitation letters were sent to potential participants by the consultant psychiatrists of community mental health teams (Phase 1) and service managers of carers support groups (Phase 2). Approval by the respective Research and Development Unit and Local Research Ethics Committee and individual consent were sought and gained. In Phase 1, 15 co-resident carers took part in in-depth interviews (initial and follow-up) in their own home (12 spouses, 2 daughters and 1 partner; 11 female, aged from 50 to 80 years old). The participants varied in gender, age, marital relationships and the duration of caring responsibility. This allowed the generation of the diversity of relevant data and facilitated constant comparisons of existing and incoming concepts and categories (Strauss & Corbin, 1998). Follow-up interviews were conducted for member checking (Charmaz, 2006; Holloway & Wheeler, 2002). The first author (interviewer) took issues (emerging concepts) that had been identified from the analysis of around two or three interviews back to each participant for their comments. In Phase 2, 21 co-resident carers were recruited for five focus groups through carers support groups within four social services districts. Thirteen participants were female and the participants’ ages ranged from 40 to 90 years old. Fourteen people were resident carers and seven participants were ex-carers whose relatives had either died or entered a nursing home. Ex-carers were included as this group was in a better position to offer insights into the later stages of caregiving at home. This was consistent with theoretical sampling and data generated through this process helped to contribute to further refinement and saturation of concepts (Charmaz, 2006; Strauss & Corbin, 1998).

### Data collection

In-depth qualitative interviews were carried out at participants’ own home in Phase 1 to gain an understanding of how they engaged their relatives in activity. Interviews allowed the participants to co-create an agenda with the researcher, to respond more fully and for the researcher to gain a greater flexibility in interacting with the participants (Gillham, 2005). Questions covered in the interview included: how carers involved their relative in activities that were considered beneficial to the person; whether carers felt that they could influence how their relative engaged in activities; what concerns they had; and how they would like to be supported during the process of engaging their relative in activities. Key categories were identified related to activity engagement, highlighting how carers’ perception of activity engagement of their relative and their own strategies changed over time. The details of the emerging themes can be seen in a previous publication (Chung, Ellis-Hill, & Coleman, 2008).

In Phase 2, focus groups were conducted in a familiar environment (locations where carers attended carers and a training centre within a local community hospital) in order to ensure easy access (Stewart, Shamdasani, & Rook, 2007). Focus groups enabled the researcher to collect and generate rich data (Fontana & Frey, 2008) from a number of people in a cost effective way and the participants had more time to reflect and to recall experiences as well as to stimulate opinion from others (Barbour, 2007). The aim of this phase was to conduct member-checking by asking the members of the focus groups questions including: to what extent carers felt that the activity patterns of their relative had changed since they first noticed their relative had memory difficulty, whether they could recognize themselves in the themes developed in Phase 1 (emergent fit) and to see if the findings made sense to them so as to refine the emerging concepts and categories from Phase 1 of the study and to facilitate the process of saturation of core categories for theory development.

### Data analysis

All interviews and focus-group discussions were audiotape-recorded and transcribed verbatim, including speech hesitations such as “umm” and “err”, to illustrate the ways that participants conveyed their messages; a dash (—) was used to indicate pauses. Names of people and places were removed for reasons of anonymity. Transcripts were entered into the Nud*ist 6 computer software package to ease the management of such a large amount of data. Each transcript was read and re-read many times and coded to develop concepts further until all core concepts were identified. These concepts were developed by constant comparison until no new categories emerged (Strauss & Corbin, 1998). Concepts were discussed within the team to seek alternative interpretations, which enhanced the qualitative data analysis. Line-by-line open coding provided a basis for constant comparison and development of concepts from data because it allowed studying actions and events within the incoming data. Axial coding was used to explore variations in patterns in the data and to further develop their characteristics in terms of properties and dimensions (Strauss 1994; Strauss & Corbin, 1998). The researchers kept an open mind about the data and avoided using any framework in a rigid manner. Selective coding was used at later stages of the analysis when core categories were being identified. This coding was more conceptual and focused (Strauss & Corbin, 1990, 1998).

The rigour of the research was enhanced in several ways. These included the first author meeting regularly with the other two authors to discuss the developing themes to check for alternative interpretations. She also sought out “negative instances or contradictory cases” in relation to the development of analytic ideas (Mason, 1996, p. 94). These case analyses provided sources to compare the similarities and differences between existing data and contradictory data; to check the extent to which it contradicted the working analysis; and to develop further the variations of the concept (Charmaz, 2006; Holloway & Wheeler, 2002). A field diary was used to facilitate continuous reflections during the process of data collection and analysis and to “trigger the researcher’s memory” (Chenitz, 1986, p. 76). Memos were used to keep a record of various components including: the analysis, identification of the characteristics of concepts and categories; questions raised during the process of analysis; the development of concepts and categories; and directions for further data collection in this grounded theory study. The use of memos enabled the organization of retrievable data for sorting and cross-referencing (Strauss & Corbin, 1990, 1998; Charmaz, 2000). It also provided an audit trail for the development of a theory in a systematic manner. Throughout the research process, the first author adopted a reflexive approach on how the “self” was used as a tool in the research process (Finlay, 2006), taking into consideration her professional background and how previous knowledge might have had an influence on the research process, including the way the data was collected and analyzed (Lincoln & Guba, 2000).

## Key findings

This paper will focus on discussing one key theme that emerged from the findings. This was that despite their relative’s significant loss of cognitive and functional ability, carers continued to recognize and support their relative’s agency in order to enhance personhood. The relationships between activity engagement, agency and personhood of those with dementia were intertwined. This section will discuss everyday activity engagement and the strategies carers used to recognize and support their relative’s agency. Pseudonyms are used throughout.

### The nature of everyday activity engagement in carers’ lives

There were two key aspects: (1) the felt importance of maintaining a sense of stability and continuity in daily life and (2) and the all-encompassing nature of supporting everyday activity and agency.

### Importance of everyday activity as a means to maintain some sense of stability and continuity in daily life

Routines and meaningful activities were one way of maintaining some stability and continuity, and were of fundamental importance for the carers. In the face of their relative’s progressive memory decline, carers considered it very important to maintain stability in their relationship by supporting their relative’s sense of control (or agency) through occupations that still had some value or meaning for their relative. Family carers did not just “look after” and “care” for their relative, they continually set and adapted the everyday activities for their relative. By encouraging and supporting the usual activities and routines of their relative as far as possible, the carers maintained their relative’s (and to some extent their own) sense of self and personhood. They created a link to the “usual” previous life that they had led with their family members (spouses, parents and partners) and created a sense of continuity with the past. This is an important aspect in anyone’s life (McAdams, 1993; Sarbin, 1986), but especially so in the face of the unusual events and the life changes brought about by cognitive decline. This could be illustrated in the quote from Susan (Phase 1):

My husband used to work in the high street, I think he probably missed the people coming to and fro… for some reason he likes to go down to the town…I think that is habit…I send him a little notes, and then he goes off and fetches them—I mean sometimes he got the wrong things but as long as it is something he is used to—that’s his routine.

Over time, the sense of agency of those with dementia was gradually challenged, they lost the ability to control their environment because of cognitive deterioration, and their carers took on responsibility for making things happen, acting according to their relative’s wishes. This can be seen in a quote by Mavis (Phase 2), who set up the environment for her husband to engage in activity and was guided by her perceptions of her husband’s intention:

You have to take on board what your partner wants to do and just guide them, you know, even to make a cup of tea—You know, if I stood in the kitchen and perhaps my husband would like to make us a cup of tea, you would agree that he couldn’t find the spoon, to find the tea bag, to find the kettle, to pour the cup…you need to offer support and guide them through.

Through supporting self-initiated everyday activities, carers provided relatives with an opportunity to act as they wished and to implement their chosen activities with support. This promoted a sense of autonomy in them by respecting their wishes.

### Activity engagement was a process that required constant decision making by carers

Involving relatives in everyday activities was all-pervasive and was not just carrying out a variety of tasks, but was part of a complex pattern of daily interactions requiring constant decision making.

All the carers attempted to encourage their relative to take an active part in everyday activities that had specific meaning to that person, both in their past and present life situation. The activities that carers supported were largely related to self-management, household management and entertainment. The types of activities that carers talked about ranged from navigating for their relative when driving, helping with paying bills, taking a short holiday break, going out for a walk, watching television together, listening to music, assisting with toileting and persuading their relative to have a regular bath. The list of activities was endless and sometimes mundane. In order to ensure that each of these activities was meaningful and appropriate to the specific needs of their relative, carers made constant decisions in four key areas: (1) setting meaningful goals for activities, (2) identifying changes (retained abilities) in their relatives’ performance of them, (3) developing subsequent adjustments to accommodate changes in the relative’s ability and (4) negotiating with them. The nature of this decision making was complex and constantly evolved during the course of caregiving. This was especially so when the family carers felt increasing difficulty in communicating with their relative concerning the latter’s wishes and preferences. An example of this is illustrated by Lilian (Phase 1) below:

In the beginning, when I said to my husband, “You are not really able to work out your accounts, are you?” He said, “Oh yes, I am”, and he’d get quite annoyed—But I mean, he went into the newspaper shop—I thought he was capable of going and buying a Radio Times…and I showed him the picture on the front—and—I said, “Go in and ask for one of this”. He said, “Fine.” Well, one of the girls served him and he gave her £75, just like that. They’re 45p. Luckily they phoned me up and said, “We made him put it back in his purse.”…So I’d to persuade him not to put too much money in his purse—say £10, and then if he wants any more he’ll have to ask me. In the beginning, he was reluctant. Now, he sort of doesn’t mind.

### The strategies used by family carers to support agency

The strategies that carers used to support agency were wide ranging and included: being non-judgemental; facilitating a sense of worth; taking calculated risks; maintaining the continuity of their relative’s identity; enhancing a sense of connection with their relative’s role and identity; using enjoyable activities; preventing inactivity and attending to the bodily source of the agency. These will be discussed in more detail below. The association between positive aspects of caregiving, agency and identity preservation, as well as the consequences of lack of support for carers and following loss of agency of their relative, will also be discussed.

### Being non-judgemental

Initially carers often went along with or agreed to something that they may not have been completely sure of in order to help their relative maintain, as far as possible, their pre-existing role and responsibility within any usual routine activity. This was often seen as a necessary adjustment to support and respect a family member who was going through some “bad days”. This can be seen in the quote below by Rob (Phase 2), who talked about how he accommodated his wife’s problem in the beginning:

When you first notice the little things…you don’t really want to sort of draw their attention to it because at the early stages you still think…I will get you over this sort of thing. Although perhaps deep in your mind you don’t realise it is, but you don’t want to bring it to their attention so that it sort of knocks their confidence or self-esteem back, you know.

By gradually taking over or compensating for their relative’s role with or without their realisation, many participants began to re-establish the boundaries of involvement with their relative at the same time maintaining some continuity in their relative’s routine and relationship. Most of the participants considered their relative’s wishes to make decisions for themselves even though they may have perceived that such decisions were unwise at the time.

### Facilitating agency by matching the goal of an activity with their relative’s retained abilities

Carers promoted their relative’s sense of success by enabling them to maximize their retained ability, which, carers considered, linked with the past self and routine activity of their relative before the onset of dementia. Through engaging their relative in a modified activity, carers facilitated a sense of worth in their relative. This could be illustrated in the quote from Liz (Phase 1):

My husband will go to the shop, the shops that he uses that he knows, but I will give him one thing written on a piece of paper. If I give him more than one he might manage to—but he may get confused and he certainly can’t remember…I have tried not to take away from him anything that he possibly could do.

Hence, stability was also maintained by controlling the parameters of an activity so that their relatives could successfully continue routine activities/roles that were usual for them. This was felt to enhance a sense of continuity of self and security for their relative.

### Taking calculated risks

Often, carers encouraged agency by developing a strategy that enabled their relative to take part in “risky” activities that their relative initiated. In such a situation, carers felt that the benefit outweighed the risk. Through engaging their relative in activities of their own choice, carers promoted a sense of independence and autonomy in their relative. This could be illustrated by a quote from Lorna (Phase 2):

My husband used to go out with the dog and he would be gone perhaps for three hours, and the dog always brought him home…I suppose he was just wandering…I used to worry like hell all the time he was out, but I didn’t want to take that little bit of independence away from him…I thought it would be good for him because his concentration was such that you couldn’t get him to do anything.

Some carers continued to help their relative carry out certain daily activities even though they may have been considered by others as risky (e.g., unsafe driving, inappropriate use of electrical appliances). They tried hard to get the balance right to enable a sense of autonomy and agency. For example, Joy (Phase 2), following the confirmation of a diagnosis of dementia given to her partner, was reluctant to cooperate with a medical consultant who advised her to stop him driving, she said:

The doctor asked my husband’s opinion, on how he felt about driving. And he said, “Well although I am still capable of driving perfectly alright I am frightened that if there is an accident, because I have got Alzheimer’s, I will get the blame even if it is not my fault.”…He did in fact change his mind and drive a bit after that when we were out in the country. I said, “Yes go for it”, and then we would get to a main road and I would take over again.

This reflected that, even after the diagnosis, many carers continued to respect their relative as an agent who was capable of making decision and could negotiate for their own needs. In Joy’s case, she realized that her husband missed his driving very much because he used to go on holidays with her in his car until he obtained the diagnosis. Hence, she was prepared to compromise her own needs.

These examples illustrated that taking risks was an inevitable aspect of carers’ daily lives. This was often at a cost to carers in terms of their own psychological and emotional wellbeing.

### Maintaining agency through the continuity of their relative’s role and identity

Often carers supported an activity, even if it was not perceived to have been “successful” in terms of outcome, because they felt it maintained some stability in terms of continuity of identity. Through engaging their relative in activities that aimed at enhancing a sense of identity, carers enabled their relative to express themself in a particular way that reflected the latter’s value and interest. This in turn enhanced a sense of agency and personhood. This can be illustrated in this quote from Carole (Phase 1):

My father is a gardener and I have a patio with pots so…I let him do whatever he likes and then I go round afterwards and sort it out again because he does some weird things now’…he’s still got the mind that he had before—it just doesn’t always show.

It is interesting to see how Carole stated, “my father is a gardener”. This was despite the fact that her father had been experiencing memory loss for many years. By maintaining her father’s routine in gardening she was also maintaining her father’s identity as a gardener. She enabled her father to act according to his will and to use his remaining abilities to carry out a familiar activity within a supportive environment. Through taking the action to go round afterwards and sort things out things in the garden for her father, Carole showed an awareness of the need to adapt the environment in order to minimize its demand on her father’s performance; and to enable her father to achieve a sense of productivity and satisfaction. There was a sense of wishing to reaffirm a sense of capacity and meaningful purpose in everyday living in their relative.

As can be seen, the perceived benefit of activity engagement often focused on supporting those with dementia in taking part in the process of meaningful doing, not just producing a tangible end product.

### Evoking agency by using enjoyable activities

Carers often tried to support enjoyable activities that still held a value for their relative. Even when severe cognitive decline was apparent, carers tried to respond to their relative’s wishes (or positive choices) and to promote their wellbeing by adjusting the physical and social environment; thus maintaining feelings of stability and connection with the past. For some carers, this could mean that, despite memory and functional loss, they could enable and share a sense of fun and enjoyment through doing things which were still meaningful to their relative, as illustrated in a quote from Gill (Phase 1):

My husband liked to watch the television, but erm, he couldn’t follow a story, so I wouldn’t put on a programme that had a continuous story because he’d be losing the plot…but something—very old programmes—sort of comedy, you know. He liked that, and it is funny…but I knew it was no good putting on, you know some murder mystery because he wouldn’t know what it was about. So he wouldn’t enjoy it—I could leave him there on his own for a while.

It seemed that, in the face of progressive cognitive decline, the need for carers to maintain a sense of enjoyment and happiness in their relative became increasingly important. Hence, carers attempted to identify meaningful activities that prevented their relative from feeling a failure and being distressed by demanding experiences.

### Promoting agency through preventing inactivity

Carers faced enormous challenges when their relative was not interested in any activities that kept either their mind or body active. They considered inactivity to be an unhealthy state, which could lead to rapid deterioration in their relative’s wellbeing. They felt it necessary to find strategies to continuously engage their relative in activities even as dementia progressed, as Ann (Phase 1) said:

I asked my husband if he wanted to do something, or go out, he just said, “No, I’m all right here”…Well, I felt I ought to stimulate him a bit more, but err, he just sits there all day and doesn’t want to be stimulated…If he doesn’t sit he will be lying and sleeping…so, I talk with him, we talk together…talk about programmes probably, and events, family events.

Being inactive was considered by carers to be a state of stagnation. Carers perceived that, in such a state, their relative ceased to act on their own volition or to express who he or she was. This created a barrier for carers, preventing them from connecting with the past self of their relative and challenging the continuity of their relative’s and their own agency. The link to the past or the person they knew appeared to be lost. Carers felt that this had an adverse impact on the relationship with their relative and so they made an effort to prevent their relative from sitting idly by encouraging some kind of action as a way of expressing themselves. This in turn enabled carers to support their relative to act on their wishes and to relate to them in a meaningful manner. Lillian (Phase 1) said:

I try to keep my husband as active as possible, because I believe that if you sit down and let it happen, you stagnate…he’s not allowed to stagnate, which I think is helpful to him.

It appeared that recognition of the continuity of the previous identity and agency of those with dementia could be best understood when a person was engaging in meaningful activities with the support of a carer who endeavoured to connect their relative’s past and present unique characteristics. This process, whereby carers sustained continuity of the current self of their relative, through bringing back the past self when the person was no longer able to do that alone, has been described by Jennings (2009) as “memorial personhood”.

### Attending to the bodily source of the agency when verbal ability reduces

It seemed evident that as family carers experienced increasing difficulty in communicating verbally with their relative, they attended more to the bodily source of agency of their relative as a means to help them understand the wishes and preferences of their relative. Such bodily sources may manifest themselves as nonverbal (e.g., affection, restlessness, hostility and anger) or verbal behaviours (e.g., verbal aggression). Carers often found clues in these behaviours to make the link between their relative’s present behaviour and their past routines, habits and preferences. Carers created tactics to understand the bodily source of agency of their relative. This can be seen in the quote below by Norman (Phrase 2), who said:

My wife would sit in her arm chair with a chest of drawers and magazines and newspapers and, she loves, rather than sit forward she loves to take the papers, tear them apart—then, fold and refold, fold and refold, and it keeps her occupied…you know, you do study your loved one very intently…they are slumping like that, they really want something to occupy their hands…Their mind is going back to when they were a wife you know what I mean, putting things away, folding, ironing, and if she is doing something she thinks she is doing the housework.

Implicit in this quote was that some carers tried to find the symbolic meaning of their relative’s action as a way to make sense of how they could support their relative to engage in activities that appeared to be meaningful to the individual. By doing so, carers also maintained a connection with the old self and meaningful relationship with the relative.

## Association between positive aspects of caregiving, agency and identity preservation

Caring for a relative with dementia can be a very difficult experience due to the loss of personhood and relationship as well as the related responsibilities. Carers faced enormous challenges when their relative was not interested in any activities that might keep either their mind or body active. The findings of this study showed that maintaining previous identity through encouraging activities and reaffirming agency of their relative could benefit the carers’ psychological wellbeing. This was shown in several ways: firstly, as carers’ lives and sense of self were intimately entwined with that of their relative, the more they maintained the “usual” life of their relative, the more they could maintain the stability of their own daily life and routine and less distressing for them, as shown in the earlier example by Susan who continued to support her husband to “go down to town” and “fetch something” from the shops.

Secondly, despite the stresses and strains from caring responsibility, many carers continued to enjoy moments when they experienced shared activities with their relative, as Tony (Phase 1) said:

We are able to get out and of course…my wife can use a walking stick and she can walk out to the car with a walking stick and walk from the car to one of the benches and then I either get the folding chair out for her or the three-wheeler, so she is able to have a bit of activity. And then of course we sit on the bench and we have an ice-cream cornet. We sit there with a cornet in our hands like a couple of kids, licking our ice creams. That is fun.

By taking his wife on a ride in a car to a familiar location, this carer achieved a sense of being relaxed and sharing companionship with his wife. There was also a sense of having a pleasurable experience in terms of making the right decision to engage his wife in a positive experience.

Some carers also encouraged activities that supported and maintained intimacy such as singing or dancing, as Nora (Phase 2) said:

I try to encourage my husband to dance and sing. I have a limited amount of success. He stands up and I put my arms around him, I know he is not very steady but he remembers what he has to do more or less and I do a lot of singing you know, to cheer myself up and I will say come on, you know this.

Implicit in this quote was that some carers attempted to identify familiar and safe activities that still had value to their relative as a way of maintaining a sense of connection, bringing pleasure to both.

## The consequences of lack of support for carers posed a risk to the maintenance of the agency of their relative

Carers attempted to continue to engage their relative in daily routine activities that enabled them, to as far as practicable, to respect their will and exercise their sense of control. Nevertheless, many acknowledged that they often felt uncertain of how to behave or respond over a range of everyday situations and found themselves struggling with the everyday management of the everyday care, even though their relative had received a diagnosis. This is reflected in this quote by Julie (Phase 1):

At first when the consultant psychiatrist said about the dementia…I thought “Ah! I’ve got a label…Now I can understand why my mother is behaving like this.”…But then it was like, that didn’t help me at all, because it didn’t explain, really. No, no. And it wasn’t like, you know, she’s got this physical thing, and means that this is how she will behave, and how she will progress, and this is what we can do, you know, this is what we can do to help…and I’m trying to manage it, and as time’s going by, I’m doing it less and less well.

Engagement in their relative’s everyday activities was a time-consuming and demanding process. Many carers worked in isolation. The findings also suggested that carers themselves often did not receive any helpful feedback from others (e.g., family or professionals) in the decisions that they had made. Hence, they often live with a lot of uncertainty and had to learn by trial-and-error. Many were living with a sense of criticism from others. As Linda (Phase 1) said:

The staff in the day centre said, “you must fetch him [my husband] and take him home.” I thought if he is sensible enough to walk home, let him! To me, I believe in rehabilitation…he gets into a habit where he’s got used to doing what he used to do [walk home from a day centre by himself], and then suddenly he isn’t allow to do it, it’s like…the staff are worried thinking why can’t me come and pick him up, you know.

This carer explained that she used to worry a lot when her husband first insisted on walking back home from the day centre on his own. The journey took about 45 min from an isolated country house through a country lane (a journey he was familiar with before prior to the diagnosis). On numerous occasions, she had tried to persuade her husband not to go out on his own without success. As a result, she had to give in to her husband’s argument and supported him to carry on walking to the property on foot. Hence, she tried to comfort herself that by supporting her husband to do what he wanted to do, she helped him get some physical exercise and therapy at the very least. Nevertheless, she resented the judgment of her by staff concerning her perceptions of risk-taking with her husband’s daily routines. Moreover, she worried that she was blamed for the perceived negative consequences of her decision making.

Even though carers faced a sense of uncertainty and criticism, many carried on developing strategies to support their relative, demonstrating how important activity engagement was for them in their lives. The findings showed that carers had determination and perseverance and devoted the time to facilitate their relative’s activity, highlighting their conviction that there was a fundamental need to maintain everyday routines and connections to the past.

As John (Phase 1) explained below, even though he received a range of outside resources to support him to care for his wife at home, he felt that he had to devote all his time, day and night, to the care of his wife because the social services care package did not provide sufficient cover for his wife’s agency needs:

Well the carer [home care assistants] comes in at 8am–9am. She gets my wife up, gives her a shower, and gets her dressed. That helps me a lot you see; but from then on, it is all over to me you see and I have to do everything…We have to carry on until something happens, you know. Until I can’t do it anymore…But she would do the same for me if the situation was reversed. I am sure she would look after me as best she could.

Carers’ decisions related to their caring role was often motivated by love, passion, reciprocity and interdependency and positive relationships between the past and present. Nevertheless, family carers often faced the challenge of whether they were making the right decisions with limited support from professional services.

It is worth noting that those carers who were caring for a relative with severe loss of cognition and functioning were often able to give accounts of episodes of positive interactions with their relative, for example, dancing and singing with them and validating their “usual” behaviours with personal meaning. Hence, by recognising the uniqueness of their relative’s individual characteristics, carers played a key role in maintaining the personhood and continuity of their relative’s identity and agency.

## Discussion

Agency is crucial to the promotion of wellbeing and the delivery of person-centred care (Jennings, 2009; Kitwood, 1993). This study has shown that family carers play an important role in sustaining the sense of autonomy and control (agency) of their relative through engaging them in daily activities at home and in the community. It has built on Kitwood’s (1993, 1997a, 1997b) work, focusing on a domestic rather than institutional setting, broadening the understanding of the processes involved in maintaining agency and personhood through activity engagement. With the trend towards inclusive community approach in dementia care and partnership working with family carers, the findings have implications for the creation of dementia-friendly communities across the country, and these will also be discussed.

Kitwood (1993) stressed that a state of wellbeing and the personhood of those with dementia is related to their agency. He described a person with dementia as an agent. He was concerned that in Western culture (in the 1990s) when a person has been diagnosed with dementia, s/he was often stigmatized and considered to have a lesser status due to their reduced cognition and functional ability. It was believed that an enabling person-centred environment and positive relationships would promote a sense of agency, social confidence and hope in those with dementia (Kitwood & Bredin, 1992). In recent years, with increasing number of people with dementia and the trend towards community care, the creation of dementia-friendly communities in society has been seen as an important strategy to overcome the stigma and isolation associated with dementia (e.g., cognitive decline and negative behavioural symptoms of persons with dementia) (DH, 2015; Alzheimer’s Disease International, 2012). It is envisaged that in such a society, individuals (especially those in early stages of dementia) would be encouraged to have choice and control in their daily lives and to engage in their daily activities within a supportive person-centered community (DH, 2012). The family carers in this study provided empirical evidence of how people with dementia can be supported in their agency and maintenance of identity through everyday activities. The use of positive interaction and relationships was evident not only in the earlier phases, but also in the later phases of caregiving (even though often lasting for only a short duration). Through engaging their relative in meaningful routine activities, carers recognized the nature of their relative’s past self, history, values, desires and current ability and they took action to develop corresponding strategies that reaffirmed their relative’s agency and personhood. The successful strategies used by the carers to reaffirm agency and identity maintenance can be adopted to guide the development of training materials that can also be shared with other informal carers. This in turn enables those living with dementia to stay connected and involved in their local communities. Such strategies include identifying enjoyable activities, maximising retained ability, facilitating a sense of worth and choice, supporting positive risk-enablement activities, attending to the bodily source of the agency and providing a nurturing environment (i.e., physical, social and psychological). They could also be used to support the development of strategies for practitioners and care workers in health, social and voluntary care sectors. This would then help to build the community capacity for an inclusive dementia community, where people living with dementia feel valued and understood (the UK Prime Minister’s Challenge on Dementia, DH, 2012). This in turn may challenge societal attitudes to dementia and promote individuals’ confidence to manage their everyday life. It will also be important for practitioners and care workers to learn about and appreciate the positive strategies used by family carers so that they feel more supported in their activities.

Engagement in activity is essential for health and psychological wellbeing (Perrin, 1996; Vernooij-Dassen, 2007) and is considered a fundamental human right for a human being (Mozley, 2001; Perrin, May, & Anderson, 2008). The consequences of being inactive lead to psychological and behaviour problems including restlessness, agitation and withdrawal (Kitwood, 1990; Scherder, Bogen, Eggermont, Hamers, & Swaab, 2010). The findings of this study showed that inactivity was a key barrier to carers being able to connect with their relative. Carers considered the need to keep their relative active through everyday meaningful activities as a means to support the continuity of their relative’s identity and agency. Moreover, this study also showed in detail how carers continuously developed strategies to engage their relative in order to meet a range of their relative’s needs including functional, psychosocial, emotional and environmental needs of the person at home. The use of activity engagement was evident throughout the caregiving journey. This is despite the fact that carers felt constantly challenged by the complex and changing circumstances, and so broadened the understanding of non-pharmacological approach involved in a non-institutional setting. It is worth noting that family carers, without formal training, are taking on an enabling role, which used to be considered as a clinical role by trained staff.

This study showed that activity engagement was a process that required constant decision making for carers. Carers felt constantly challenged by the complex and constant changing circumstances. This included behavioural difficulties manifested by their relative during activity engagement. This study has highlighted that providing mechanisms of support as a family member was very different from doing so as a paid carer (the target audience for Kitwood’s original mechanisms of support). The basis of Kitwood’s person-centred approach is that when positive interactions have been applied consistently, and over a long period, many psychological needs of the person with dementia are met (Kitwood, 1997a, 1997b). Care staff are encouraged to show unconditional acceptance, for example, being generous, forgiving and expecting no reward (Kitwood, 1997b). In contrast, this study of carers in the home environment highlighted a key difference: family carers often felt uncertain of how to act/behave or respond over a range of everyday situations and they were unsure whether the approaches they used were appropriate or not. This finding is consistent with the studies of Keller et al. (2007) and Vikstrom, Borell, Stigsdotter-Neely and Josephsson (2005). In addition, this study showed that carers found it difficult to work out appropriate types of positive interactions (during activity engagement) with their relative at different points of caregiving. Moreover, the carers’ home situation changed a great deal over time (compared to a care setting). Family carers, in the main, offered unconditional acceptance to a great degree; however, they found it very hard to behave in this way for 24 hours a day, 7 days a week. Many felt isolated in their role as a carer. In some circumstances, the notion of positive interaction (at all times) placed a heavy responsibility on family carers and made them feel guilty. This challenged carers’ own sense of identity and confidence in their ability to sustain their role and adversely affected carers’ own agency, personhood and wellbeing.

This shows that it is crucial to support family carers in meeting the continuing activity needs of those with dementia if person-centred care in dementia care is to be adopted fully in a home setting. This could be achieved through recognising the agency and identity work that carers have already been carrying out effectively and using this as a basis for understanding. Using a non-judgmental approach is crucial as it can be seen that carers already feel under pressure, often feel like failures and are less likely to try new strategies that they have found useful if they feel they are going to be judged. If support for carers is not provided, it is possible that they will lose touch with the needs of those with dementia (or unwittingly deprive them of their autonomy and dignity) because they themselves do not have the energy, confidence and/or skills to assess and act within the situation. This in turn may lead to incidents of ignoring, disempowerment, objectification, banishment and invalidation, termed Malignant Social Psychology by Kitwood (1990, 1993), and potentially carers’ abusive behaviours (Cooper et al., 2010). Moreover, the personhood of those with dementia would be compromised. There is clearly a need for family members to be supported in learning how to be able to adapt behaviour. The successful strategies adopted by family members in this research will form a basis for developing such principles. This is important if dementia policies (Alzheimer Society, 2014; DH, 2009, 2010, 2012, 2015) are to be implemented to enable persons to “live well” with dementia in their own home, within the context of person-centred care.

It is crucial for practitioners to recognize the individual strategies that carers are currently using, to check with the carers if they feel that the strategies they using are useful or not, and to be able to suggest other strategies (proposed by family carers) that could lead to a successful intervention. It is also important to help carers recognize those strategies that might lead to failure or frustration. A model that highlights the strategies that family carers use successfully at different phases of dementia care has been drawn up by Chung et al. (2008) and may serve as a useful reference point. The model of activity engagement could be used to highlight and explain to the carers the processes they are going through and the importance of promoting their own activities and mental health. Moreover, it enables carers to recognize their own strengths and vulnerabilities and therefore develop realistic strategies to support their own needs. Ultimately, this would enhance carers’ own sense of agency as well as ensuring that that of their relative remains as high as possible.

This study provides some evidence for and highlights the importance of professionals working collaboratively with family carers, especially when assessing the actual performance of those with dementia. This is because family carers often have practical experience about the embodied knowledge of their relative. Moreover, such tactic knowledge enables professionals to gain insight into the mechanisms of support that family carers offer to their relative and how some family carers maintain a connection with the old self of and meaningful relationship with the relative. This insight and understanding is crucial if professionals are to work collaboratively with carers to develop a personalized intervention plan for those with dementia, as promised in the government policy (e.g., the National Dementia Strategy [DH, 2009] and the Prime Minister’s Challenge on Dementia [DH, 2012, 2015]) and to support carers to enable their relative to be “a full and equal citizen”, as advocated in the Carers Strategy (DH, 2008). In addition, the insight into how family carers support the agency and personhood of individuals with dementia would help raise awareness and understanding of what these individuals can still do and contribute with the support of their communities. This in turn facilitates the development of dementia-friendly communities, which is a major element of the UK Prime Minister’s Challenge on Dementia since its launch in 2012.

### Strengths of the study

The research was based on the data from family carers’ own stories through in-depth interviews and focus groups. Participants in the interviews and focus groups were very experienced carers who had been through the prolonged process of engaging their relative in everyday routine activity and seeking appropriate and relevant help. They were therefore in a position to talk about what might be the most appropriate strategies for activity engagement. Hence, the strength of the study is the knowledge and insight gained from the carers of this particular group regarding their perception of activity engagement.

Also, this study made it easy for carers to talk about positive aspects of their experiences. It has been suggested that research on the positive aspects of caring is limited (Cohen, Colantonio, & Vernich, 2002; Nolan, Lundh, Grant, & Keady, 2003; Searson, Hendry, Ramachandran, Burns, & Purandare, 2008). The advantages of exploring the positive experiences of caregiving have been discussed in the literature (e.g., Andrén & Elmståhl, 2005; Carbonneau, Caron, & Desrosiers, 2010; Kramer, 1997): firstly, carers wanted to talk about such experiences, as doing so led to a feeling of pride in their ability to meet challenges in their new role and hence to a feeling of self-worth. Secondly, such knowledge helps practitioners to work more effectively with carers by identifying positive outcomes and carers’ satisfaction. Thirdly, this line of inquiry in caregiving research has the potential to provide information for the development of theories of carers’ adaptation and psychological wellbeing.

### Limitations of the study

The transferability of this study is limited by the fact that the study has focused on co-resident carers’ decision making in their engagement of people with dementia at home, from the onset of dementia to the point when they relinquished their caregiving responsibility, either to hospital admission or residential care, and how carers played an important role in sustaining the sense of autonomy and control (agency) of their relative through engaging them in daily activities. Due to the fact that they were a self-selecting participants (i.e., very dedicated to the wellbeing and activity needs of their relative at home), the experience of such a group of co-resident carers might not be considered to be typical of co-resident carers. However, from the researchers’ clinical experience it was felt that although these carers were not specifically skilled, they were definitely committed to care for their relative and wished to enable their relative to stay at home for as long as practical. The researchers feel that this would be the case for most carers of people with dementia. The results may not be applicable to non-resident carers and relationships within residential care due to the different contexts involved, as Strauss and Corbin (1998) stated that “reproducing social phenomena can be difficult because it is nearly impossible to replicate the original conditions under which data were collected or to control all the variables that might possibly affect findings” (p. 266). Despite that, Holloway (1997) and Lincoln and Guba (2000) pointed out that researchers could enhance the transferability of findings of a study by adopting strategies such as detailed contextual descriptions of the research and explicit details of the research process. This in turn will facilitate the reader to make judgements as to whether the findings of the present study could be transferred to relevant contexts or similar participants (Kvale & Brinkmann, 2009). The researchers therefore hope that the findings concerning the characteristics of the co-resident carers add to the understanding of the diversity of carers’ needs and an understanding of how activity engagement could be used to enhance identity in a person-centred manner. It was worth noting that on their departure many participants in the focus groups thanked the researcher for giving them an opportunity to take part in the discussion group and acknowledged that it was helpful for them to talk about their experience. A couple of them also thanked her for the way in which the group discussion was handled, as talking about caring experiences was a very emotional and sensitive subject for them. Hence, it would appear that the group participants felt secure in exchanging their opinions with others in a group setting. This ultimately increased the usefulness and quality of focus group data (Stewart et al., 2007).

In conclusion, the findings support many of Kitwood’s ideas and have provided empirical evidence of and illuminated how the agency and personhood of those with dementia is maintained by family carers at home. This study provided further insight into how carers played an important role in sustaining the sense of autonomy and control (agency) of their relative through engaging them in everyday activities and the centrality of the carer-relative relationship in dementia care in family care setting. Carers play a key role in creating an enabling environment to support their relative’s agency in many ways and develop strategies that are constantly reassessed and modified over time. Lack of timely and ongoing support for carers could ultimately pose a risk to the maintenance of the agency of persons with dementia. The successful strategies used by the carers to reaffirm agency can be adopted to guide the development of not only assessment and intervention strategies for people with dementia, but also training resources for carers (both paid and unpaid) to enable those with dementia to stay active and connected and feeling in control in dementia-friendly communities.
